# Description and circadian rhythms of *Chandlerella sinensis* Li, 1933 (Nematoda; Onchocercidae), with remarks of microfilariae effects on the host health

**DOI:** 10.1017/S0031182024000738

**Published:** 2024-06

**Authors:** Rasa Binkienė, Ralph E. T. Vanstreels, Mélanie Duc, Rasa Bernotienė

**Affiliations:** 1 P. B. Šivickis Laboratory of Parasitology, Nature Research Centre, Vilnius, Lithuania; 2 Karen C. Drayer Wildlife Health Center, University of California, Davis, CA, USA

**Keywords:** bird, filarioid, histology, mosquito, pathogenicity, phylogeny, sequences

## Abstract

During investigation of common linnet *(Linaria cannabina)* blood using the buffy coat method one bird with microfilariae in the blood was found. The morphometric description of adult worms corresponded to the *Chandlerella sinensis.* This species was found for the first time in common linnets. DNA sequences of *cox1* and *28S* gene fragments of adult worm recovered during necropsy was identical to that from the microfilariae in the bird blood. Phylogenetic analysis of the *cox1* gene fragment clustered this parasite with *Chandlerella quiscali*. Histological examination revealed the presence of microfilariae in the lumen of small capillaries and other blood vessels in different organs, but no inflammations were notice. The greatest number of microfilariae was in the lungs. Even if there was no inflammation, but vessels associated with the lungs were markedly distended with blood, parabronchial walls were thickened and, in some cases, almost completely obstructing the lumen. The large number of microfilariae in lungs indicates possible disturbance of gas exchange in the lungs adversely affected the ability of the bird to exercise and made breathing difficult at rest. The investigation of circadian rhythm of the microfilariae showed that *C. sinensis* microfilariae in blood of common linnet were more numerous at night and morning and less numerous at midday. The survival rate of mosquitoes infected with *C. sinensis* microfilariae was significantly lower than that of uninfected mosquitoes.

## Graphical abstract

**Figure:**
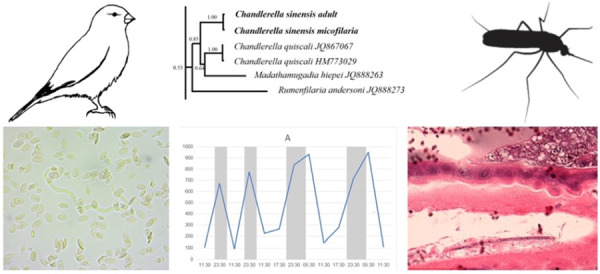

## Introduction

The filarioid nematodes of the family Onchocercidae Leiper, 1911 (Spirurida) infect a wide range of hosts, including amphibians, reptiles, birds and mammals (Anderson, 2000; Lefoulon *et al*., 2015). Out of 16 genera of bird-infecting onchocercid, *Chandlerella* Li, 1933 is one of the largest genus and comprises 26 species of parasites (Bartlett, 2008). The adult parasites of *Chandlerella* can be found in different organs and cavities like blood and lymph vessels, heart, liver, spleen, kidneys, lungs, brain, mesentery, celomic cavity, air sacs, connective and subcutaneous tissues (Sonin, 1966; Bartlett and Anderson, 1980*a*). Adult female filarioid nematodes produce microfilariae that enter the bloodstream and can be transmitted by arthropod vectors. Microfilariae are usually considered non-pathogenic (Bartlett, 2008), but some studies have reported on the pathogenicity of adult filarioids for anatids, psittacids, pigeons, passerines and emu (Weinmann *et al*., 1979; Paster, 1983; Allen *et al*., 1985; Kummerfeld and Daugschies, 1989; Law *et al*., 1993; Cole, 1999; Bartlett, 2008; Huang *et al*., 2017; Atawal *et al*., 2019). While extensive research has been conducted on the effects of other avian blood parasites such as *Plasmodium* Marchiafava and Celli, 1885 and *Haemoproteus* Kruse, 1890 on the survival of infected vectors, studies on the pathogenicity of avian microfilariae to their vectors are lacking (Anderson *et al*., 2000; Valkiūnas *et al*., 2014; Bukauskaitė *et al*., 2016).

A wide variety of blood-sucking arthropods (fleas, flies, lice, ticks, mites, biting midges and culicine mosquitoes) has been described as vectors of onchocercidean nematodes (Anderson, 2000; Bartlett, 2008; Bain *et al*., 2013). However, most of the research on the life cycle of filarial nematodes have focused on parasites of mammals, and the intermediate hosts of only nine avian-infecting onchocercidean species from almost 170 are known. Among them, the life cycles of 3 species of *Chandlerella* have been studied in North America, and biting midges (Ceratopogonidae) of the genus *Culicoides* Latreille, 1809, were found to be involved as their vectors (Robinson, 1971; Bartlett and Anderson, 1980*a*; Anderson, 2000).

Several studies have investigated aspects of biological rhythms of parasites such as the periodicity of host–parasite systems, the relationships between the biotic environment of the parasites and the rhythms of vectors, the immune response of the host, the level of hormones and oxygen in the blood of the host (Hawking, 1967, 1975; Pichon and Treuil, 2004; Reece *et al*., 2017). There are different patterns of parasite rhythms, and microfilariae usually show 24-h rhythms being most numerous in the peripheral bloodstream during some periods of the day and absent or less numerous at other times (Hawking, 1967, 1975; Anderson, 2000). Since two hosts are involved in the life cycle of onchocercidean nematodes, the juxtaposition of the circadian rhythms of both hosts may increase the chances of transmission and development of these parasites. This rhythmic interaction has been well described for human parasites. For example, the diurnal increase in parasitaemia of *Loa loa* (Guyot, 1778) (human loiasis) microfilariae coincide with the activity of *Chrysops* Meigen. 1803 flies, while *Wuchereria bancrofti* (Cobbold, 1877) and *Brugia malayi* (Brug, 1927) microfilariae exhibit increased parasitaemia during the night, which coincides with flying activity of their mosquito vectors (Hawking, 1975; Anderson, 2000).

In recent decades, molecular biology has been instrumental to elucidate the life cycle of heteroxenous parasites of vertebrates and invertebrates (Criscione *et al*., 2005). However, the genetic markers are known only for a few species of avian-infecting onchocercidean nematodes making it difficult to detect and identify the parasites in these hosts.

The aim of the work was: (i) to describe the filarioid species parasitizing common linnets (*Linaria cannabina*), (ii) to elucidate the circadian rhythm of the microfilariae of this parasite, and (iii) to investigate the pathogenicity of microfilariae for both intermediate and definitive hosts.

## Materials and methods

### Material collection, fixation and staining

Twenty-three common linnets were caught at Ventės Ragas Ornithological Station, Lithuania (55°20′28.1″N 21°11′25.3″E) during spring migration in May 2021. The birds were captured with mist nets, zig-zag traps and large funnel type traps. They were ringed, identified and examined at study site by ornithologist. Approximately 20 μL of blood was taken by puncturing the brachial vein and collected using heparinized capillary tubes. A few drops of blood were used to prepare thin blood smears, and the remaining blood was maintained in the capillary tubes to be examined using the buffy coat method (Chagas *et al*., 2020).

One infected bird was used to determine the daily periodicity of microfilariae (see below). The same infected bird and one non-infected bird were used as blood meals for mosquitoes to investigate their survival. The non-infected individual was released after blood sampling. After investigation of periodicity and mosquito survival, the infected bird was euthanized by decapitation in the evening after blood feeding of mosquitoes and immediately examined. Organs and other tissues were placed in Petri dishes containing 0.9% saline solution, and the parasites were recovered using dissecting needles and pipettes under stereomicroscope. The subcutaneous tissues, joints of leg and wings, brain, eyes, heart, connective tissues of trachea and oesophagus, lungs, liver and body cavities were examined for the presence of adult filarioid nematodes according to Binkienė *et al*. (2021). Adult live nematodes were examined under the microscope in 0.9% saline solution, and then stored in 70% ethanol. For light-microscope examination, they were cleared with glycerine (Singh, 1948), one female and one male were mounted in Berlese's medium to facilitate examination of copulatory apparatus. Blood was collected from the liver and lungs and stored in 96% ethanol for molecular analysis. Blood smears from the brachial vein, liver and lungs were fixed with methanol, stained with Giemsa and examined under light microscope (Binkienė *et al*., 2021). The following measurements were obtained from microfilariae using the DeltaOptical DLTCam Viewer 3.7.8301 software: total body length, headspace, tail length, the maximum width of body, and distance of fixed points from anterior or posterior extremity; fixed point values were expressed as percentages of the total body length.

Parasite species was identified using identification keys and species descriptions (Sonin, 1966; Olsen and Braun, 1976; Bartlett and Anderson, 1987*a*). Voucher specimens were deposited in the Nature Research Centre (NRC) in Lithuania (accession numbers ECOHELMI 1164-1172).

### Histological examination of organs

Fragments of the brain, heart, lungs, liver, kidneys, pancreas and pectoral muscles of the infected individual were collected and fixed in 10% formalin. After 24 h, the organs were washed with distilled water and placed in 70% ethanol. In the laboratory, the organs were embedded in paraffin wax, sectioned at 4 μm, stained with haematoxylin-eosin and examined by light microscopy (Bacha and Bacha, 2000). Histological slides were deposited to NRC (accession numbers ECOHELMI 1215-1228).

### Phylogenetic analysis

Parasite DNA was extracted from bird blood, microfilaria-infected bird tissues and adult nematodes according to Stunžėnas *et al*. (2011), with a minor modification according to Petkevičiūtė *et al*. (2014). Partial sequences of mitochondrial cytochrome c oxidase I (*cox1*) and nuclear 28S rDNA (28*S*) genes were amplified through polymerase chain reaction (PCR) (Binkienė *et al*., 2021) using primers COIintF and COIintR, and Nematode 1 and Nematode 2, respectively (Casiraghi *et al*., 2001; Sehgal *et al*., 2005). Amplification products were evaluated by 1% agarose gel electrophoresis and both strains were sequenced using the same primers and Big Dye Terminator v3.1 Cycle Sequencing kit and ABI PRISM™ 3100 (Applied Biosystems, Foster City, USA). Sequences were edited and aligned to create a consensus sequence using the BioEdit software (Hall, 1999). Sequences obtained in this study were deposited in GenBank (accession No. OR346687, OR346688, OR350920).

Phylogenetic analyses were performed for sequences of each gene separately using Geneious Prime 2023.2 software [https://www.geneious.com]. MrBayes plugin v3.2.6 (Huelsenbeck and Ronquist, 2001) was run in Geneious for 3 million generations, with the sample frequency every 100 generations. The first 25% trees were discarded as a burn-in step. A total of 27 sequences (2 from this study) and 21 sequences (2 from this study) were used in phylogenetic analysis of the *cox1* and *28S* genes respectively, comprising sequences from other filarioid genera from birds (*Chandlerella* Yorke and Maplestone, 1926, *Eufilaria* Seurat, 1921, *Splendidofilaria* Skrjabin, 1923, *Pelecitus* Railliet and Henry, 1910, *Eulimdana* Founikoff, 1934, *Cardiofilaria* Strom, 1937, and *Aproctella* Cram, 1931), mammals (*Wuchereria* Silva Araujo, 1877, *Rumenfilaria* Lankester and Snider, 1982, *Mansonella* Faust, 1929, *Dirofilaria* Lubimov, 1935, *Onchocerca* Diesing, 1841, *Breinlia* Yorke and Maplestone, 1926, *Burgia* Buckley, 1960, *Loa* Stiles, 1905, and *Setaria* Viborg, 1795), reptiles (*Madathamugadia* Chabaud, Anderson and Brygoo, 1959*)* and *Filaria latata* Chabaud and Mohammad, 1989 as outgroup.

### Microfilarial circadian rhythm

In order to evaluate the periodicity of microfilariae changes along the day, an infected bird was kept 4 days in cage, with provided water and food *ad libitum*, at room temperature (~22°C) and natural light-dark period (17:7 h). During this period, blood (approximately 15 μL) was taken each 12 h (11:30 and 23:30 h) in the first and the second days and each 6 h (5:30, 11:30, 17:30 and 23:30 h) in the third and the fourth days. Three blood smears were prepared each time and the blood was investigated using the buffy coat method as described by Chagas *et al*. (2020). The buffy coat and adjacent plasma were transferred to a microscope slide and covered with a coverslip (18 × 18 mm). The entire preparation was examined and microfilariae were counted under microscope at 200× magnification. Live nematodes were photographed with a Zeiss PrimoStar light microscope equipped with an AxioCam ERc 5s camera (Carl Zeiss). After the microscopic examination, the coverslip was removed and a thin smear was prepared, fixed and stained following the same procedures as for blood smears (slides deposited in NRC accession numbers ECOHELMI 1202-1214). The *t* test for independent samples was used to compare parasitemia (microfilariae/μL of blood) between different times of the day.

### Investigations of mosquitoes

Wild caught mosquitoes were blood fed from one infected and one uninfected common linnet. This was done at the study site (Ventės Ragas). Engorged mosquitoes were collected using methods described by Valkiūnas (2005). Briefly, the bird was held by hands covered with nitrile gloves close to bushes and exposed to mosquito bites in the evening (from 6 PM). When several females began taking a blood meal on a bird's head the head with feeding insects was placed into an unzipped insect cage (approximately 15 × 15 × 15 cm). The engorged insects fly off after the blood meal into the cage, the bird was taken out and the insect cage zipped. This procedure was repeated several times until not less than 30 insects were placed in the insect cage. The zipped insect cages with engorged insects were transported to the laboratory and maintained in standard conditions (20 ± 1°C, 70 ± 2% RH and L:D photoperiod of 17:7 h). Mosquitoes were fed with 5% saccharose solution. Only fully engorged females were used in this study. Dead and moribund mosquitoes were collected from cages several times per day, starting 12 h post-infection (hpi) until 14 days post-infection (dpi). Mosquitoes were identified according to Becker *et al*. (2003). The Kaplan–Meier estimator was used to evaluate differences in mosquito survival among different groups.

Some dead mosquitoes were examined for observation of microfilariae: they were placed in 0.9% saline solution, and their thorax and abdomen were dissected under stereomicroscope. Tissue and midgut were covered with a coverslip, and parasites were examined under a light microscope at 200× magnification. Additionally, the full bodies of 5 dead mosquitoes were fixed in 10% formalin and proceeded for histological examination as described for the organs of the bird host (slides deposited in NRC accession numbers ECOHELMI 1229-1332). All mosquitos that survived to 14 dpi were euthanized with ethanol and dissected.

## Results

Examination of bird blood using the buffy coat method revealed that one common linnet was highly infected with microfilariae. In total, 52 adult nematodes were obtained post-mortem from the liver blood vessels. The morphology and morphometric parameters of adult worms corresponded to *Chandlerella sinensis* Li, 1933 (Table 1). Since the common linnet is a new host for *C. sinensis*, a brief description of the nematode is given below.

**Table 1.** Morphometric parameters of adult nematodes of *Chandlerella sinensis* found in different hosts (*Linaria cannabina*, *Dendrocitta formosae sinica* Stresemann, 1913 and *Dicrurus forticatus* (L)) according to our findings and previous references

	*L. cannabina* (present study)		*D. formosae sinica* (Li, 1933)^a^		*D. forticatus* (Chabaud *et al*., 1964)	
Parameter	Male (*n* = 6)	Female (*n* = 7)	Male	Female	Male	Female
Body total length, mm	10.1–12.2 (11.2)	14.7–20.8 (17.1)	15–18	23–25	22	29
Body maximum width	188–235 (213)	243–350 (287)	140–170	230–250	150	200–345
Body width at nerve ring	65–90 (80)	65–102 (82)				
Body width at vulva		98–140 (116)				
Body width at cloaca	62–90 (70)					
Oesophagus total length	548–697 (614)	526–690 (608)				
Oesophagus total width	37–56 (46)	37–48 (43)				
Muscular oesophagus length	145–189 (165)	133–198 (156)	140–150	160–190	200	230
Muscular oesophagus width	16–26 (21)	17–25 (20)				
Glandular oesophagus length	403–510 (457)	380–499 (451)	500–510	500–620	500	620
Glandular oesophagus width	37–56 (46)	37–48 (43)				
Vagina length		570–792 (707)				500
Left spicules length	63–77 (70)		70–80		100	
Left spicules width	12–17 (14)					
Right spicules length	49–60 (55)		50–60		80	
Right spicules width	8–12 (10)					
Distance						
AE to nerve ring	92–144 (118)	80–117 (106)	100–120	110–130		
AE to vulva		230–370 (300)		360–430		480
PE to anus	233–287 (246)		200–230		300	
PE to ovary		24–367 (117)				

*Notes*: AE, anterior extremity; PE, posterior extremity.

All measurements are given in micrometres, unless indicated otherwise.

a Type description.

### Description

**Figure 1.**
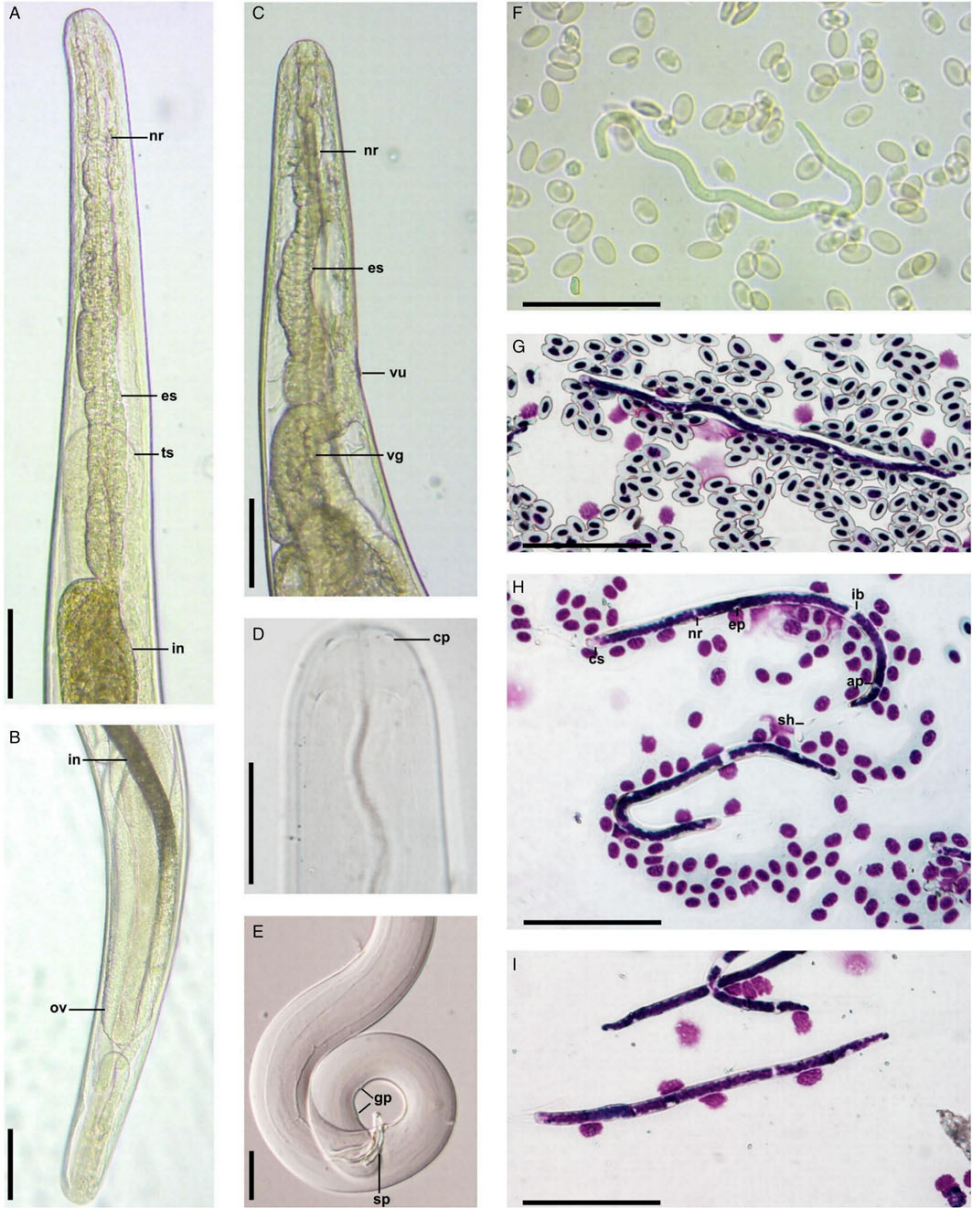
*Chandlerella sinensis*: male anterior extremity (live worm) (A); female posterior extremity (live worm) (B); female anterior extremity (live worm) (C); female anterior extremity (mounted in Berlese's medium) (D); male posterior extremity (mounted in Berlese's medium) (E); microfilariae in blood (live worm) (F); microfilaria in peripheral blood (Giemsa stain) (G); microfilaria in pulmonary blood (Giemsa stain) (H); microfilariae in liver blood (Giemsa stain) (I). Abbreviations: cp – cephalic papillae; ep – excretory pore; es – oesophagus; gp – genital papillae; ib – inner body; in – intestine; nr – nerve ring; ov – ovary; sp – spicule; ts – testis; vg – vagina; ap – anal pore; sh – sheath; vu – vulva. Scale-bars: A-C – 100 μm; D–I – 50 μm.

Adult nematodes are slender with slightly attenuated broadly rounded extremities and cuticle without bosses. Males are shorter than females. The oral opening is small, and the oesophagus is externally divided with the anterior extremity of the muscular portion expanded in bulb-like enlargement (Fig. 1A and C). The vagina is short and directed posteriorly, and the anus of the female is reduced (Fig. 1B). There are 2 pairs of cephalic papillae, which are not markedly protuberant but can be seen in the specimens mounted in Berlese's medium (Fig. 1D). Males have 3 symmetric pairs of postanal genital papillae: first pair 104–128 (116), second pair 60–79 (73) and third pair 36–51 (43) from the posterior extremity (Fig. 1E). Spicules are subequal and dissimilar: left spicula have well expressed manubrium and calamus, narrow tip; right spicula is shorter and of almost equal width throughout its length (Table 1). The vulva in the first part of glandular oesophagus is not marked by any prominence. The vagina gradually expands into a sac shape, runs backward from vulva and joins the didelphic uterus.

Microfilariae are short and sheathed (Table 2, Fig. 1F–I). Their body is filled with small nuclei up to the posterior extremity. G and R cells are not well distinguishable. The tail slightly narrows broadly rounded.

**Table 2.** Measurements of *Chandlerella sinensis* microfilariae from blood, lungs and liver

	Blood (*n* = 20)		Lungs (*n* = 20)		Liver (*n* = 20)	
Parameter	μm	%	μm	%	μm	%
Length	128–164		118–152		112–157	
Maximum width	4–5		4–5		4–5	
Inner body length	2–5		2–4		2–6	
Cephalic space	2–4		2–4		2–4	
Distance from AE to:						
Nerve ring	37–46	25–29	30–41	24–29	30–43	24–29
Excretory pore	50–61	34–39	41–57	33–40	41–58	32–42
Excretory cell	53–65	36–43	44–60	36–42	43–60	37–45
Inner body anterior end	82–105	62–67	76–98	62–68	75–102	63–70
Inner body posterior end	85–110	64–70	78–102	64–71	77–106	65–72
Anal pore	114–148	82–93	105–132	86–92	101–146	88–93

*Note*: AE, anterior extremity; % – values of fixed points expressed in percentage.

### Phylogenetic analysis

**Figure 2.**
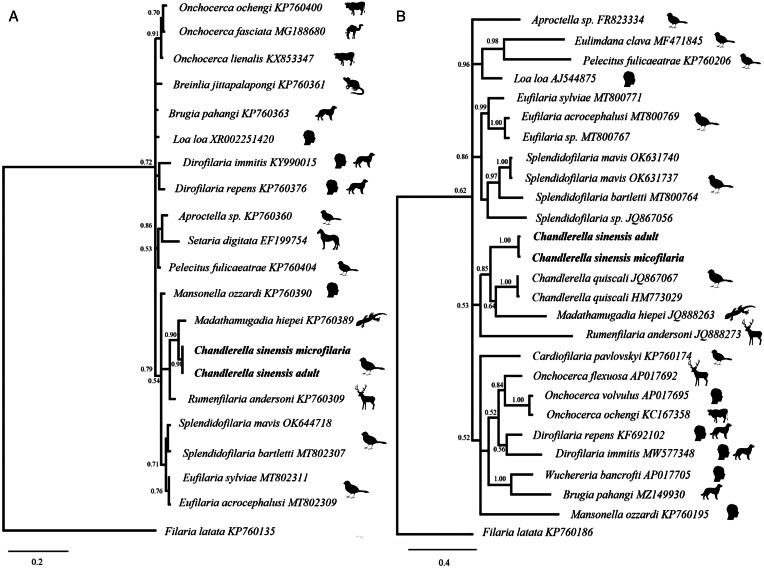
Bayesian phylogenetic trees for a segment of the *28S* gene (A) and a segment of the mitochondrial *cox1* gene (B). Sequences from this study (bolded) were obtained from microfilariae in the peripheral bird blood and an adult nematode recovered from the same bird *Linaria cannabina*. Branch lengths are drawn proportionally to the extent of changes (scale-bars are shown). Numbers adjacent to nodes represent posterior probabilities.

Two sequences, from microfilariae in the peripheral bird blood and from an adult nematode, were obtained from each the *cox1* (672 bp) and the *28S* genes (800 bp). Sequences were trimmed so that all sequences used in the phylogenetic analysis were of the same length (605 bp for *cox1* and 390 bp for *28S*). To confirm that microfilariae from the bird blood were responsible for the adult filarioid nematode recovered during necropsy, the sequences obtained from adult worm and from bird blood with microfilariae were compared. The *28S* sequences from both microfilariae an adult worm were identical, whereas a single nucleotide polymorphism was found comparing the *cox1* sequences obtained from microfilariae and from adult worms (Fig. 2).

Both gene sequences in this study were clustered in one clade with parasites from gecko (*Madathamugadia hiepei* Henring-Hagenbeck, Booker, Petit, Killick-Kendrick and Bain, 2000) and ruminants (*Rumenfilaria andersoni* Lankester and Snider, 1982) (Fig. 2A, B). Phylogenetic analysis of the *cox1* gene clustered the parasite in this study with *Chandlerella quiscali* (von Linstow, 1904) (Fig. 2B) and there were not publicly available *28S* gene sequences for *Chandlerella* sp.

### Pathology of microfilariae to the avian host

**Figure 3.**
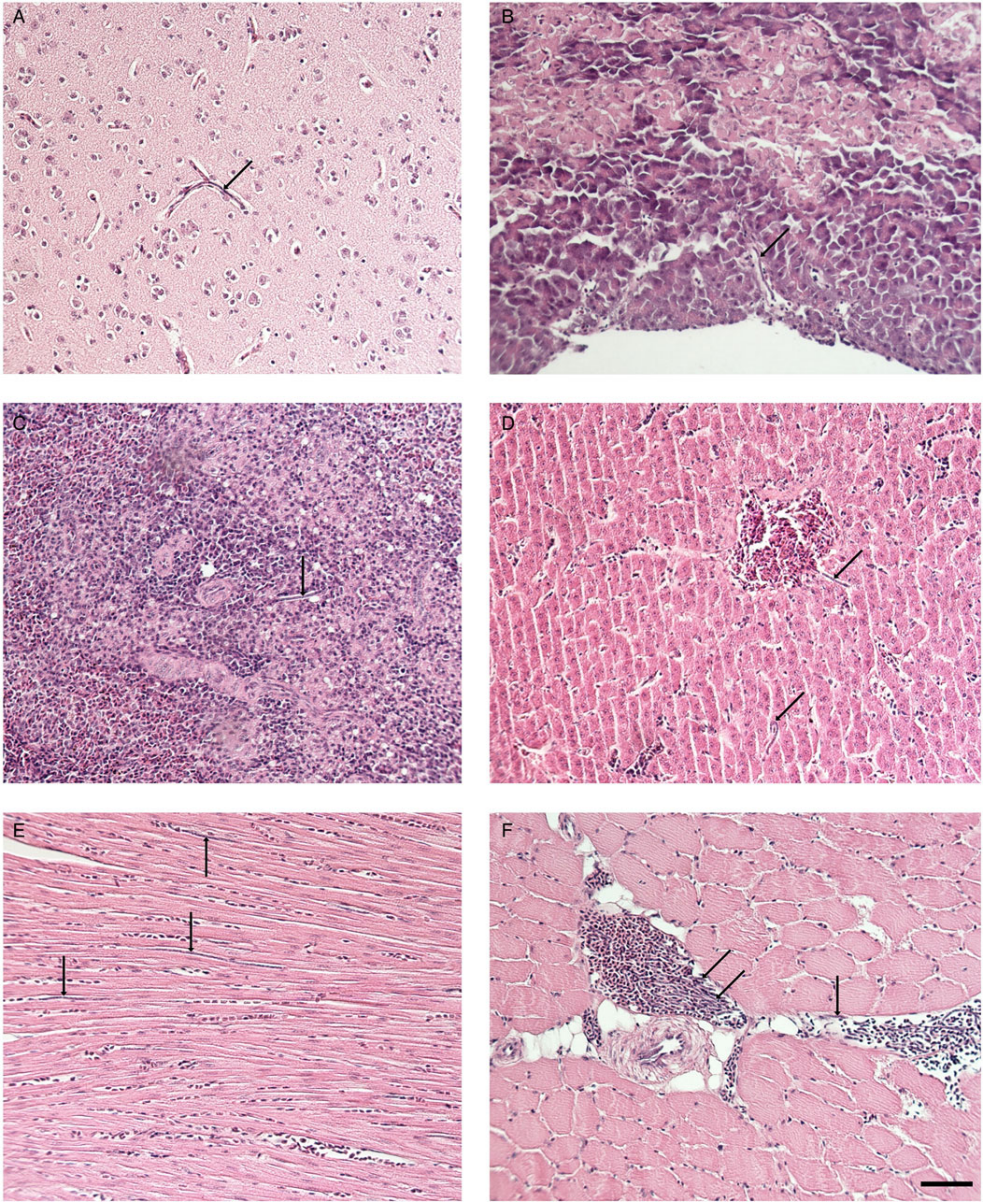
Histological section of bird organs: cerebrum (A), pancreas (B), spleen (C), liver (D), heart muscle (E), pectoral muscle (F). Arrows indicates microfilariae of *Chandlerella sinensis* in the lumen of blood vessels. Scale-bar: 50 μm.

**Figure 4.**
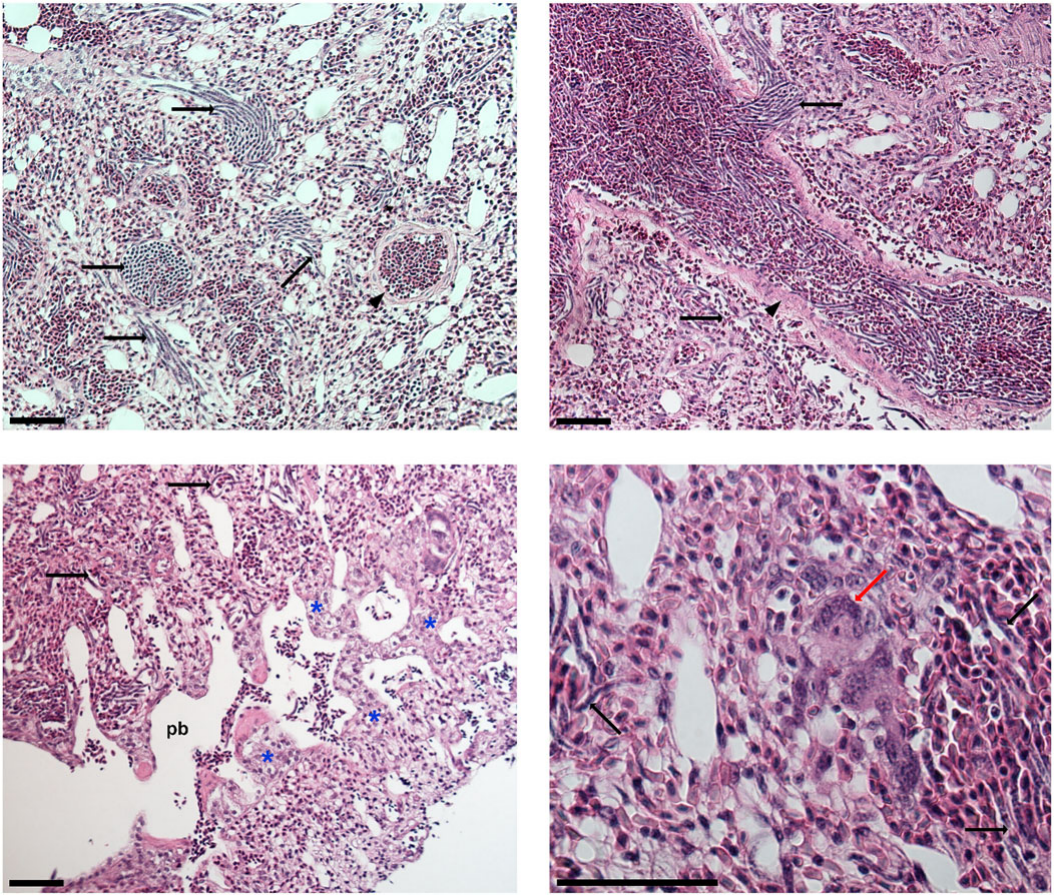
Histological section of lungs with microfilariae of *Chandlerella sinensis* infection. Indications: black arrows – microfilariae; red arrows – Langhans type giant cell; arrowheads – arteriole wall; blue asterisk – areas with an increased in the number of type II pneumocytes; pb – parabronchus. Note that only a few microfilariae are indicated, but many more are visible. Scale-bar: 50 μm.

The necropsy of the common linnet did not reveal any visible pathological changes in the internal organs. Histological examination showed the presence of microfilariae in the lumen of small capillaries and other blood vessels of the cerebrum, heart, pectoral muscles, liver, lungs and in smaller numbers in capillaries of the spleen and pancreas. No inflammatory response was seen in the cerebrum, muscles, liver, spleen and pancreas (Fig. 3). The greatest number of microfilariae was in the lungs (Fig. 4). The pulmonary parenchyma showed severe diffuse congestion, and the large blood vessels associated with the lungs were markedly distended with blood. The parabronchial walls were thickened due to the mild-to-moderate congestion and interstitial oedema. Microfilariae were extremely abundant within pulmonary arterioles/venules and, to a lesser extent, within pulmonary capillaries. In some cases, microfilariae formed parallel beams within pulmonary arterioles/venules completely or almost completely obstructing the lumen. Microfilariae did not appear to be attached to the endothelial wall. There are several instances where the microfilariae perforated the blood vessels and escaped into the air ways or the interstitium; this was often accompanied by haemorrhage. Multifocal proliferation of type II pneumocytes was also diffusely present in the lung parenchyma and giant cells (mainly Langhans type) were sporadically present.

### Microfilarial circadian rhythm

**Figure 5.**
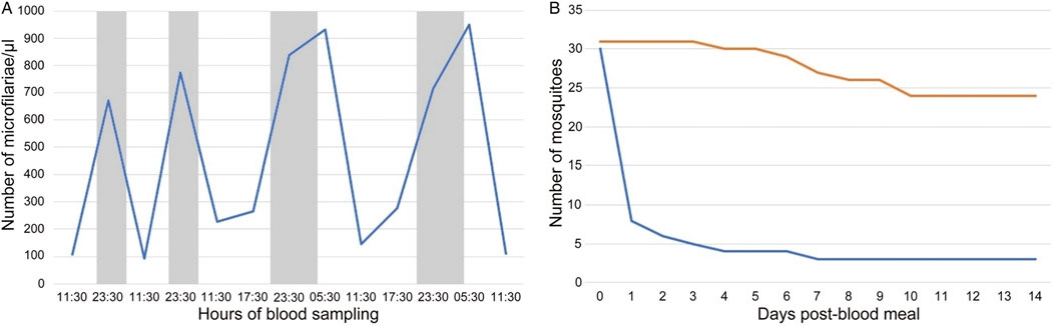
A – Circadian periodicity of *Chandlerella sinensis* parasitemia (microfilariae per μL) in the peripheral blood of common linnet (the grey field indicates the dark period of the day); B – Survival of mosquitoes fed with infected blood (blue line) and uninfected blood (orange line).

Microfilarial parasitaemia showed an apparent circadian periodicity in the peripheral blood of the studied bird. The highest parasitemia was observed at 23:30 h and at 5:30 h (Fig. 5A). Significant differences were found in the parasitemia in peripheral blood between samples collected at 17:30 h (= 271 ± 7 microfilariae/μL (mean ± s.d.)) and at 23:30 h (750 ± 63) (*t* = 2.8, *P* < 0.05) and between samples collected at 5:30 h (941 ± 10) and at 11:30 h (136 ± 48) (*t* = 19.7, *P* < 0.001).

### Investigations of mosquitoes

Mosquitoes of 3 species were collected at the study site: *Aedes vexans* (Meigen, 1830), *Ochlerotatus cantans* (Meigen, 1818) and *Ochlerotatus cataphylla* (Dyar, 1916). Thirty mosquitoes were fed with blood from infected bird (*A. vexans* (*n* = 1), *O. cantans* (*n* = 2), *O. cataphylla* (*n* = 27)) and another 31 were fed with blood from noninfected bird (*A. vexans* (*n* = 1), *O. cantans* (*n* = 4), *O. cataphylla* (*n* = 26)).

The survival rate of mosquitoes fed with blood infected with microfilariae was significantly lower than that of uninfected mosquitoes (χ^2^ = 26.8, *P* < 0.001). Of the 30 mosquitoes fed with infected blood, 22 died within 24 hpi (*A. vexans* (*n* = 1), *O. cantans* (*n* = 2), *O. cataphylla* (*n* = 19); Fig. 5B). During the dissection of freshly dead mosquitoes, 408–2119 (mean = 775, *n* = 6) microfilariae were found in the abdominal part of the body. Five mosquitoes fed with infected blood died later in the experiment (Fig. 5B, all *O. cataphylla*). Three *O. cataphylla* mosquitoes fed with blood infected with microfilariae survived until 14 dpi (336 hpi) and were examined post-mortem but no microfilariae were found. Only 6 mosquitoes fed with uninfected blood died during the 14 dpi (*O. cantans* at 96 hpi, *O. cataphylla* at 156, 168, 172, 192, 240 hpi).

**Figure 6.**
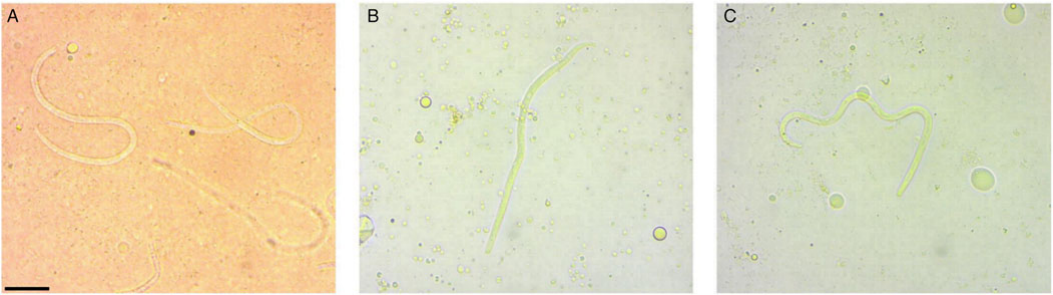
Microfilariae of *Chandlerella sinensis* in mosquitoes: A – intestine 26 h post infection, B – haemocoel 11 hpi; C – haemocoel 17 hpi. Scale-bar: 100 μm.

Examination of dead mosquitoes fed with blood infected with microfilariae showed that further development of microfilariae did not occur in them (Fig. 6), since microfilariae found in the abdomen of mosquitoes that died as late as 50 hpi were still agile and morphologically unchanged.

**Figure 7.**
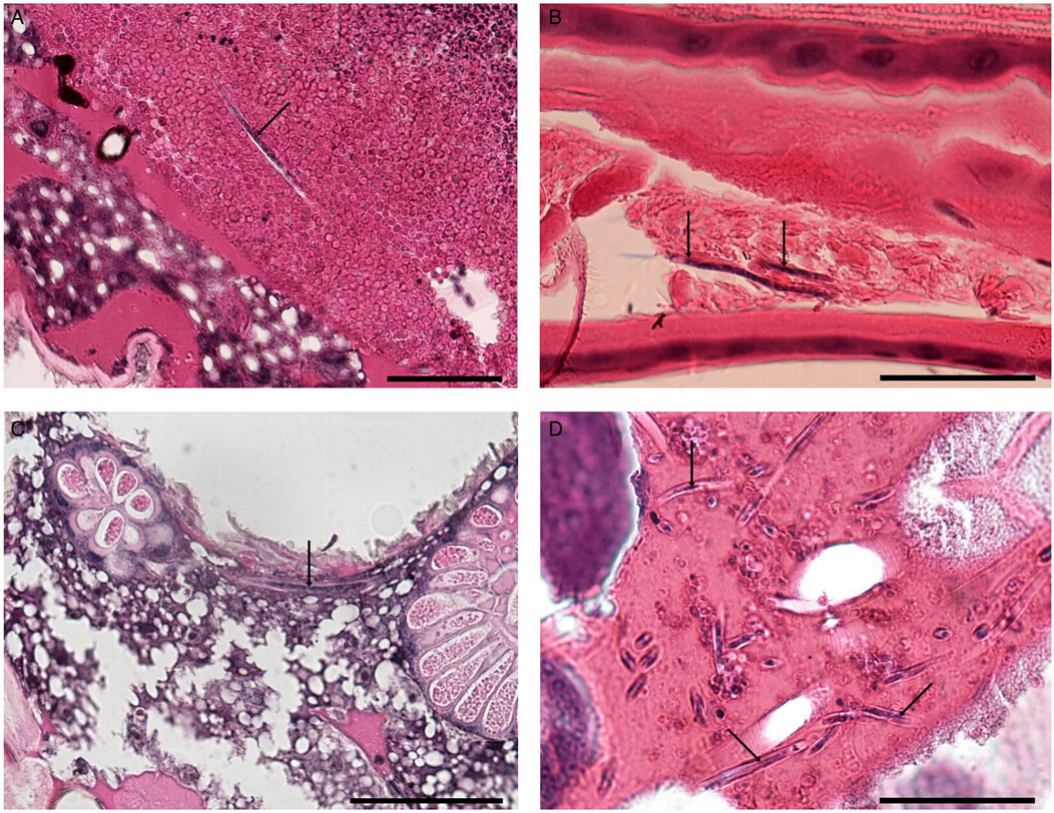
Histological sections of *Ochlerotatus cataphylla* mosquitoes fed with blood with microfilariae of *Chandlerella sinensis*: A– microfilaria in midgut; B – microfilariae in foregut; C – microfilaria in thorax between salivary glands; D – microfilariae in haemocoel, between the gonads. Scale-bars = 50 μm.

Five *O. cataphylla* that died after being fed with infected blood (deaths between 14 and 58 hpi) were fixed for histological examination. Examination of histological slides of 2 mosquitoes (22 and 38 hpi) revealed that the majority of microfilariae were located in the abdominal part in the midgut close to the peritrophic matrix, no microfilariae were found outside the peritrophic matrix (Fig. 7A). Another mosquito (26 hpi) had microfilariae in the foregut close to the peritrophic matrix and in ectoperitrophic space (Fig. 7B). One mosquito (14 hpi) had numerous microfilariae outside the midgut in the haemocoel (Fig. 7D), around the gonads and one microfilaria was present in the thorax between salivary glands (Fig. 7C). Microfilariae in the muscles of the thorax were not found.

## Discussion

Nematodes belonging to the genus *Chandlerella* have been reported from 12 orders and 27 families of birds from Europe, Asia, Africa and America. The majority of the species were described from passerine birds belonging to the families Motacillidae, Muscicapidae, Certhiidae, Nectariniidae, Icteridae, Estrildidae, Sturnidae, Oriolidae, Cicruridae and Corvidae (Sonin, 1966; Olsen and Braun, 1976; Bartlett and Anderson, 1980*a*, 1987*a*; Bartlett, 2008). To the best of our knowledge, this is the first record of nematodes of the genus *Chandlerella* in the common linnet, a passerine bird of the family Fringillidae.

The species *C. sinensis* was described from the respiratory system of corvids from Peiping, China (Li, 1933). Since the first description this parasite species was reported from corvids from Georgia, Uzbekistan, China, Tajikistan, and Madagascar (Chabaud *et al*., 1964; Chatterjee *et al*., 1965; Sonin, 1966). Chabaud *et al*. (1964) found a filarioid nematode during an investigation of the Madagascar-endemic crested drongo (*Dicrurus forficatus*, L; Passeriformes: Dricuridae). The authors considered that the morphological features of this parasite overlapped with those of *C. sinensis*, *C*. *columbigallinae* (Augustine, 1937) and *C. singhi* Ali, 1956, and concluded that it should be assigned to the earlier described *C. sinensis*. However, the specimens from crested drongo differs from *C. sinensis* described by Li (1933) by having longer spicules, asymmetric 3–5 pairs of caudal papillae, longer muscular oesophagus and bigger distance from anterior extremity to vulva. The length of organs and distances may slightly vary due to fixation and host species, but spicules are an important morphological feature in nematode taxonomy because they vary in length and shape between species, even between closely related species, and the method of fixation does not affect to spicules length (Croll and Matthews, 1977; Musah-Eroje *et al*., 2021). It is therefore reasonable to suspect that the parasite reported by Chabaud *et al*. (1964) from crested drongo likely represents a novel species of the genus *Chandlerella*, and further morphological and molecular studies are needed to confirm this hypothesis.

The morphology of *C. sinensis* bears close resemblance to that of *Chandlerella bosei* (Chandler, 1924). The original description of *C. sinensis* by Li (1933) distinguishes these species based on the arrangement of the uteri. Chandler (1924) found two distinct filarioids in the same bird and was not sure that female belonged to the same species as the male. Therefore, the females of *C. bosei* should not be considered in comparison and descriptions of the other species of the genus *Chandlerella*. In this study, we found that the main difference between males of these 2 species is the shape and length of spicules, which as previously discussed is an important feature for species identification in this group. The spicules of the *C. bosei* are ‘approximately alike’ (Chandler, 1924), while the spicules of *C. sinensis* differ substantially in both length and shape.

The spicules of *C. sinensis* in this study has morphological similarities to those of *Chandlerella columbae* (Sonin, 1966). This filarioid parasite from yellow-footed tree-pigeon (*Treron phoenicopterus*) from India was originally identified as *C. columbigallinae* (Rasheed, 1960), but later due to the morphological inconsistency was reassigned to the new species *C. columbae* (Sonin, 1966). The main differences between *C. sinensis* and *C. columbae* in this study were related to the distance to the vulva (230–430 and 446–466 μm, respectively), tail length in males (200–287 and 174–178 μm, respectively), and tail length in microfilariae (10–19 and 26–29 μm, respectively). Nevertheless, it seems that some species of the genus *Chandlerella* are morphologically very similar and can be difficult to distinguish on the basis of morphology. Further molecular analyses could help distinguishing or synonymizing the aforementioned species.

Our phylogenetic analysis clustered *C. sinensis* together with *C. quiscali* from the common grackle (*Quiscalus quiscula*), a large icterid passerine from North America (Peer and Bollinger, 2020). Lefoulon *et al*. (2015) analysed the molecular phylogeny of onchocercid worms and identified 5 strongly supported clades within the family. However, their phylogenetic analysis included only 3 species of avian onchocercid worms, which clustered together with parasites from mammals and reptiles (Lefoulon *et al*., 2015). Our study included a greater variety of avian onchocercid species, but we also could not find support for a monophyletic origin for the species that parasitize birds. Instead, our phylogenetic analysis clusters avian-infecting species together with the species parasitizing primates, ruminants and reptiles, which corroborates the interpretation that these parasites underwent multiple events of host-switching in their evolution.

The investigation of circadian rhythm of microfilariae shows that the *C. sinensis* in the common linnet are more numerous at night and morning and less numerous at midday. The nocturnal periodicity was discovered of *C. quiscali* microfilariae in common grackle from North America also (Odetoyinbo, 1960; Vaughan *et al*., 2012). It is known that majority of mosquitoes and biting midges are active during the night and twilight period (Becker *et al*., 2003; Sanders *et al*., 2012). The circadian rhythm observed in this study is therefore consistent with the interpretation that nocturnal/crepuscular increase in parasitaemia in the avian peripheral bloodstream is advantageous to the parasite in terms of increasing the likelihood of ingestion by insect vectors (Hawking, 1967).

*Chandlerella sinensis* is a host generalist and has been detected in birds of the families Corvidae, Dicturidae and Sturnidae from Europe and Asia. Filarioid worms with a wide range of host species are usually associated with non-specific feeding habits by their vectors (Bartlett and Anderson, 1980*b*). The life cycles of only 3 species of *Chandlerella* (*C. chitwoodae* Anderson, 1961, *C. quiscali* and *C. striatospicula* Hibler, 1964) have been studied in North America. These life cycles involve Ceratopogonidae midges of the genus *Culicoides* as vectors (Robinson, 1971; Bartlett and Anderson, 1980*b*; Anderson, 2000). The intermediate hosts are known for only another 6 species of avian onchocercid worms (out of 170 species known to infect birds). *Culicoides* spp. are involved in the transmission of *Splendidofilaria picacardina* Hibler, 1964, *Eufilaria kalifai* (Millet and Bain, 1984), and *Eufilaria longicaudata* Hibler, 1964 (Millet and Bain, 1984; Anderson, 2000). Furthermore, *Splendidofilaria fallisensis* (Anderson, 1954) can be transmitted by *Simulium* Latreille, 1802 flies, *Pelecitus fulicaeatrae* (Diesing, 1861) and *Sarconema eurycerca* Wehr, 1939 can be transmitted by lice of the genera *Pseudomenopon* Mjöberg, 1910 and *Trinoton* Nitzsch, 1818 (Anderson, 1956; Seegar *et al*., 1976; Bartlett and Anderson, 1987*c*). Considering that the most abundant mosquitoes at Ventės Ragas were *Ochlerotatus* spp. Lynch Arribalzaga, 1891, it seems reasonable to suspect that *Ochlerotatus* mosquitoes could be involved in transmission of *C. sinensis*. Studies on the development of *Chandlerella* spp. microfilariae in *Culicoides* spp. showed that microfilariae can be found in thoracic muscles after 12 hpi, and that after 24 hpi the parasites were sluggish; any microfilariae remaining in the digestive tract by 42 hpi were dead (Robinson, 1971; Bartlett and Anderson, 1980*b*; Anderson, 2000). Microfilariae ingested by *O. cantans* and *O. cataphylla* mosquitoes collected at Ventės Ragas did not present any apparent changes in morphology or activity. This fact shows that vectors of the *C. sinensis* in northern Europe should be not *Ochlerotatus* mosquitoes, even though these mosquitoes are abundant in May-June (Becker *et al*., 2003). As adult parasites have been found in birds of various families, but not all blood-sucking insects are involved as intermediate hosts, it can be assumed that parasites of this genus are more specific to intermediate hosts than to definitive hosts. It therefore seems likely that *Culicoides* biting midges are involved in the transmission of *C. sinensis* in northern Europe also.

In nature, insects can die from senescence or they may be killed by predators, disease and other hazards (Gillies and Wilkes, 1965; Clements and Paterson, 1981; Valkiūnas *et al*., 2014). Our results show that the ingestion of avian blood heavily contaminated with onchocercidian microfilariae can be lethal to *O. cantans* and *O. cataphylla* mosquitoes. The mosquito mortality caused by parasites has been demonstrated as the most important determinant of a species' ability to transmit vector-borne infections such as malaria, filariasis and arboviruses (Dye, 1992; Valkiūnas *et al*., 2014). After ingestion by the intermediate host, microfilariae will attempt to leave the midgut and continue their development in the haemocoel, muscles, fat body, and Malpighian tubules (Anderson, 2000). Therefore, it seems likely that the high mortality (73%) of infected mosquitoes in this study could be due to a heavy mechanical disruption of the intestines or muscles as these parasites migrated through these tissues. For instance, studies on *B. malayi* (human lymphatic filariasis) have shown that muscle cell damage tends to be more severe in artificially infected *Aedes aegypti* vectors than in *Mansonia uniformis* (Theobald, 1901), which is a natural vector (Beckett, 1971). In this context, our findings suggest that *Ochlerotatus* spp. are probably not competent hosts of *C. sinensis* and the damages made by microfilaria on these insects can be more crucial than on competent host. However, our histological analysis of mosquitoes' organs indicates that mosquito death is caused not only by mechanical damage to the gut or muscles but also by other factors influenced by microfilariae that have not yet been resolved.

Microfilariae located in the lumen of the blood vessels are generally considered as non-pathogenic in birds (Bartlett, 2008). In spite of the high density of *C. sinensis* microfilariae in the lungs of the common linnet in this study, these parasites apparently were not accompanied by a significant inflammatory cellular infiltration. The thickened parabronchial wall and vascular damage and haemorrhage are likely to have significantly impaired air-to-blood gas exchange, which presumably could have affected the bird's ability to exercise. Our pathological findings are comparable to those of a study on sick, injured or dead boreal owls (*Aegolius funereus* (L)) in Canada, wherein the extremely large quantities of microfilariae seen in the lumen of the blood vessels of some owls was considered to have significantly impaired normal circulatory dynamics and gas exchanges (Larrat *et al*., 2012). Although no behavioural studies have been carried out on birds with and without microfilariae, observations of these birds during the study showed that the linnet with microfilariae was more ‘apathetic’ and only flew when we tried to catch it for a blood sample, while the other bird started flying as soon as we entered the room with the cages.

## Data Availability

All data generated or analysed during this study are included in this published article. All newly generated sequences were submitted to the GenBank database. The voucher material (see in methods) was deposited to the Nature Research Centre, Lithuania.
